# Directional excitation of surface plasmon using multi-mode interference in an aperture

**DOI:** 10.1038/s41598-020-78594-7

**Published:** 2021-02-04

**Authors:** M. Z. Alam, Z. Yang, M. Sheik-Bahae, J. S. Aitchison, M. Mojahedi

**Affiliations:** 1 Department of Electrical and Computer Engineering, Queen’s University, Kingston, K7L 3N9 Canada; 2 Department of Physics and Astronomy, University of New Mexico, Albuquerque, NM 87131 USA; 3 Department of Electrical and Computer Engineering, University of Toronto, Toronto, M5S 3G4 Canada

**Keywords:** Electrical and electronic engineering, Nanophotonics and plasmonics

## Abstract

Plasmonics is a promising technology that can find many applications in nanophotonics and biosensing. Local excitation of surface plasmons with high directionality is required for many of these applications. We demonstrate that by controlling the interference of light in a metal slot with the adjustment of the angle of incidence, it is possible to achieve highly directional surface plasmon excitation. Our numerical analysis of the structure showing a strong directionality of excited surface plasmon is confirmed by near field scanning measurements. The proposed structure can be useful for many applications including excitation of plasmonic waveguides, nanolithography, and optical sensing**.** To illustrate its usefulness, we experimentally demonstrate that it can be used for highly directional excitation of a dielectric loaded plasmonic waveguide. We also propose a simple structure for surface plasmon interference lithography capable of providing high image contrast using this scheme.

## Introduction

Surface plasmon (SP)—the collective oscillations of electrons at the interface between a metal and dielectric can be useful for a wide range of applications ranging from communication to biosensing^1–4^. The most commonly used methods to excite SP are by the use of prisms, or gratings. These schemes are not very compact and introduce a lot of noise from the illumination source, hence they are often not suitable for nanophotonic applications. As an alternative to these traditional approaches, direct illumination through a slot in metal layer has been used as a highly localized source of SP^5^—this scheme is simple and noise free. However, the resulting SP radiates away from the hole in every direction, which diminishes its usefulness for many applications. Unidirectional and local excitation of SP has been an active area of research, and many schemes have been proposed to achieve this goal including: gratings, multiple coherent beams, partially filled or asymmetric holes^6–22^. However, these approaches suffer from a number of limitations including complicated geometries and narrow bandwidth. As a result, nonlocal excitation schemes are still commonly used in nanoplasmonics^23–31^. A compact and simple approach which provides highly directional SP excitation will be a key step towards making plasmonics a more useful technology. The improved control of near field that will be available from such a scheme may also lead to many new applications in various areas of nanophotonics.

In this work we present a very simple method for achieving unidirectional SP excitation through a metal slot. The unidirectionality is achieved by controlling the interference of the modes supported by the slot using the angle of incidence of the input light. We verify the effectiveness of this scheme by measuring the near field intensity distribution of the SP around a narrow slot in a continuous metal layer, under oblique backside illumination. The unidirectionality we achieved is much larger than predicted by previous theoretical estimates for oblique backside illumination of metal slots^22^. The proposed scheme extends the flexibility in manipulation of the near field, and can lead to various useful applications. To illustrate this, we experimentally demonstrate coupling of light to a dielectric loaded plasmon waveguide. In addition, we present the concept of a plasmonic nanolithography scheme, which is simple to implement, and provides high image contrast.

## Principle of operation

**Figure 1:**
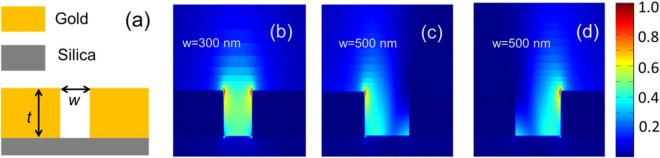
(**a**) Schematic representation of the single metal slot. (**b**) Power density profile for a single mode slot (w = 300 nm). (**c**) and (**d**) Power density profiles for a multi-mode slot (w = 500 nm ) when the two supported modes are in phase {(**c**)} and 180° out of phase {(**d**)} at the bottom of the slot. Gold film thickness (t) is 200 nm.

When a slot in a metal film is illuminated by light from the back side, the light transmission is mediated by the modes excited in the slot, which acts as a waveguide^32^. The amount of transmitted power and the modes excited on the exit side of the slot depend on the efficiency of excitation of various modes within the slot and their mutual interactions. We aim to use this phenomenon to achieve unidirectional excitation of SP. To achieve an insight into the various possibilities, we have analyzed the structure shown in Fig. 1a. The structure consists of a slot of width *w*, in a gold film of thickness *t*. All numerical results for this work were obtained using Lumerical FDTD with a wavelength of 920 nm in all cases. The material properties for gold and silica are taken from^33,34^. When the slot is very narrow, e.g. *w* = 300 nm, only the first-order mode in the metal-dielectric-metal (MIM) waveguide is supported. This mode propagates through the slot and at the end of the slot either couples into the SP modes or radiates into the free space above the metal. The power distribution for this case is shown in Fig. 1b. For a wider slot, multiple modes are supported. As an example, for *w* = 500 nm, the slot supports two modes. The direction of power flow at the exit of the slot in this case will depend on the relative amplitude and phase of the two modes. To illustrate this, we have plotted the power density profiles for the cases when the two modes are excited with equal amplitude and are either in phase (Fig. 1c) or 180° out of phase (Fig. 1d). Lumerical mode source has been used in these simulations to properly excite the modes for the analysis reported in Fig. 1. In a physical implementation, the mode excitation can be controlled by a number of ways including multiple beams^18^ or controlling the angle of excitation (proposed in this work). As can be seen from the figures, the interactions of the modes can result in highly directional power flow in the half space above the metal surface. An illustration of this principle for unidirectional SP excitation can be found in^18^. Unlike^18^ where multiple coherent beams were used to properly excite the required modes, we propose to achieve this by adjusting the angle of incidence of a single laser beam. The simplicity of our proposal can be useful for many nanophotonic applications.

**Figure 2:**
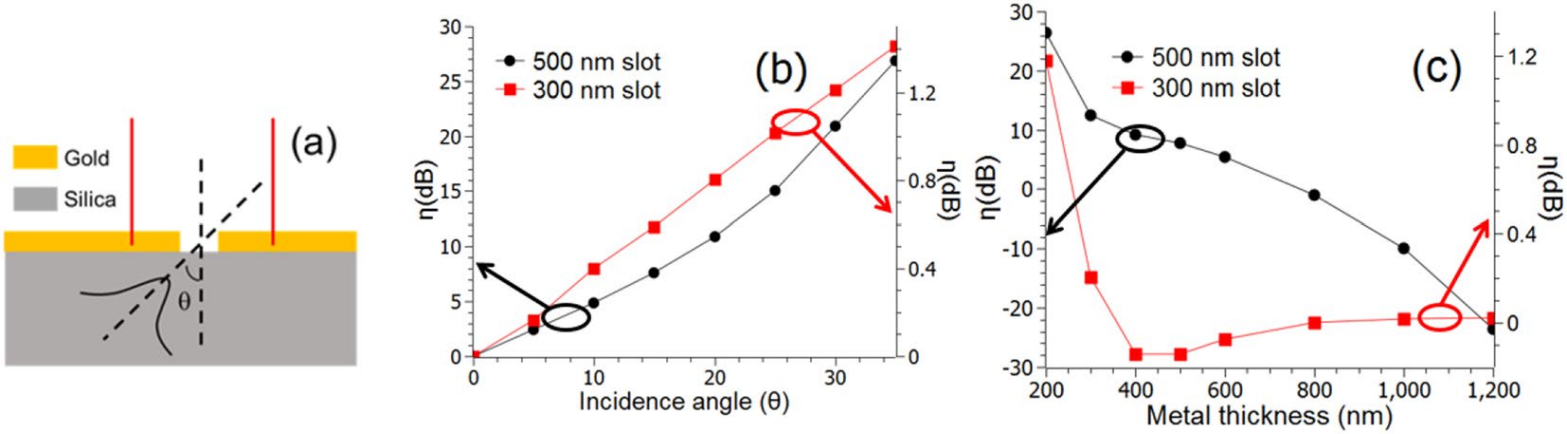
(**a**) Schematic diagram showing the simulation set up. (**b**) and (**c**) Enhancement of unidirectionality as a function of incidence angle and metal film thickness for single mode (*w* = 300 nm) and multimode (*w* = 500 nm) slots. Gold thickness for (**b**) is 200 nm, and the angle of incidence for (**c**) is 30°.

The excitation efficiency of different modes and their interaction in the slot are affected by a number of parameters including the angle of incidence, geometry of slot, and metal thickness. Therefore, it is possible to adjust the direction of power flow by controlling any of these parameters. To illustrate this, we study the effects of two parameters, i.e., angle of incidence and metal film thickness on the directionality of the SP. The simulation setup is shown in Fig. 2a. The structure is excited using a Gaussian beam from underneath at an angle θ with respect to the normal to the metal–silica interface, and the fields are recorded in simulation at equal distance on the two sides of the slot marked by the red lines. Lumerical FDTD’s built in mode expansion monitor is then used to decompose the recorded field into various modes to calculate the fraction of the power converted to SP. To quantify the directionality of the power flow, we define a unidirectionality factor (*η*) as

1 $$ \eta = \frac{{SP_{right} }}{{SP_{left} }} $$

Here *SP*_*right*_ and *SP*_*left*_ are SP generated on the right and left sides of the slot. Figure 2b shows the values of *η* as a function of incident angle for both single mode (*w* = 300 nm) and multimode (*w* = 500 nm) slots. From Fig. 2b the unidirectionality factor ranges from 0 to 1.4 dB over the range of angles plotted for the single mode slot. In contrast, η can be very large, for a multimode slot, over the same range of angles. Figure 2c shows the directionality as a function of metal thickness at a constant incident angle (θ = 30°) for both single and multimode slots. It is clear that *η* is not significantly affected by the metal thickness for a single mode slot but varies widely for a multimode slot. These results can be explained from the physical picture presented in Fig. 1c,d. As the two modes having different effective mode indices propagate through the slot, the relative phase between them changes, and as a result, the power distribution at the exit of the slot also changes. Therefore, it is expected that the metal film thickness has a significant effect on the enhancement in the case of wide slots. At this point, it is relevant to consider the discussion in^22^, where the authors have concluded that for an oblique incidence the metal film acts as a geometrical shadow. While such a shadowing may be the dominant cause of unidirectionality in the case of single mode slot, it fails to explain the behavior observed in the multimode case. From Fig. 2c it can be observed that high level of unidirectionality can be achieved toward the right side or the left side, even when the beam is only tilted to the right. Also *η* varies strongly with metal thickness in the multimode case but remains relatively unchanged in the single mode case. These trends are difficult to explain using the notion of geometrical shadowing alone but can be easily explained with the concept of multimode interference. Guided by this concept we have, for the first time, observed that very large value of *η* can be achieved by simple adjustment of beam tilt without the need for any complex geometry employed in many previously reported structures.

**Figure 3:**
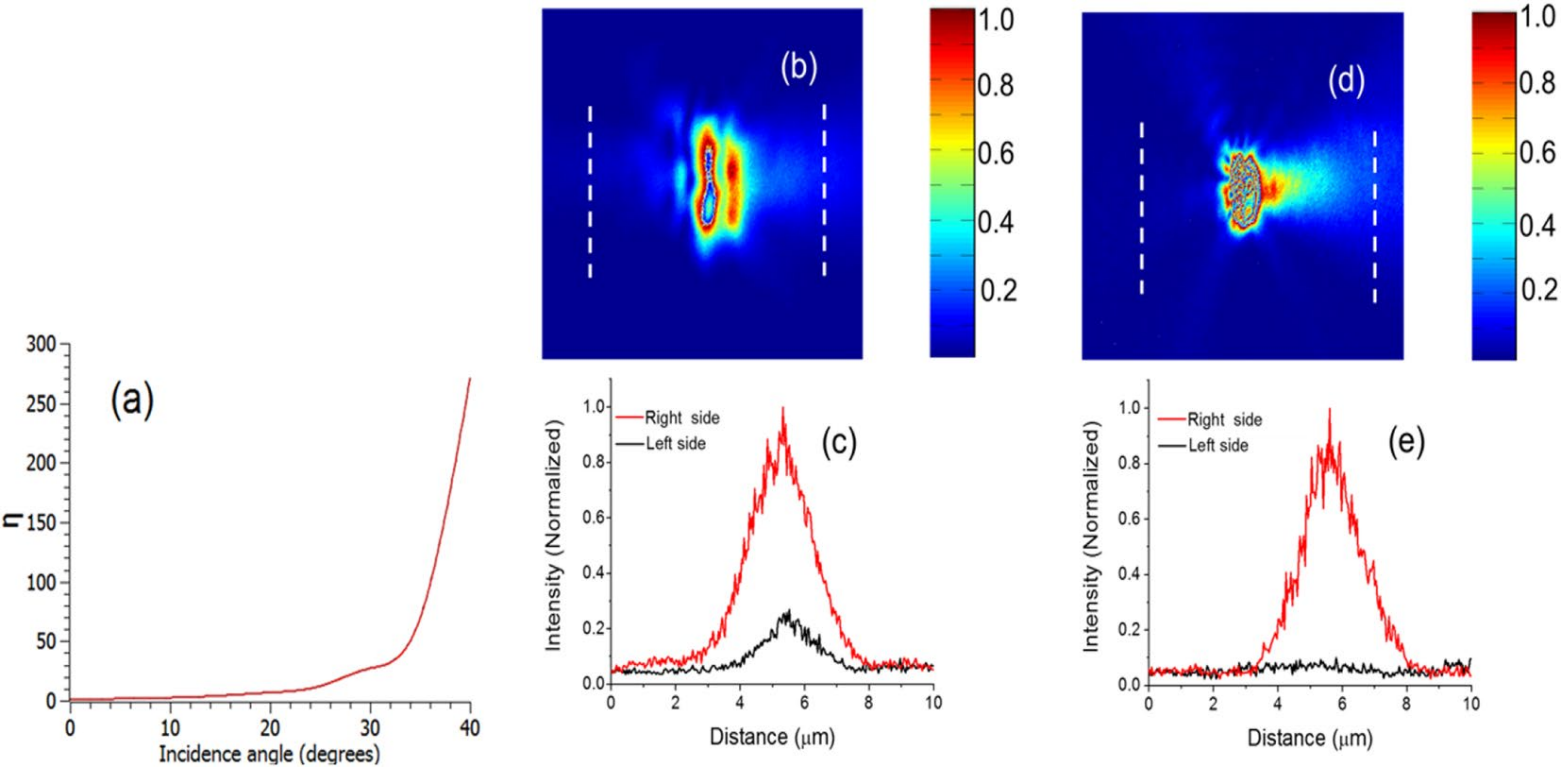
(**a**) FDTD simulation results for unidirectionality factor (*η*) as a function of the beam tilt for a rectangular hole of dimensions 2 µm × 0.5 µm. (**b**) and (**c**) Near field measurement results for θ = 15°. (**d**) and (**e**) Near field measurement results for θ = 42°.

The example of the two-dimensional structure discussed above illustrates that excitation of various modes to achieve unidirectional excitation is possible by simply adjusting the angle of incidence of incident light. The principle is very general, and can be extended to three dimensions, which are more relevant for practical applications. To verify this, we fabricated a series of isolated holes in a 200 nm thick gold film and carried out near field measurements. Details of fabrication and measurements are provided at the end of the paper. Here we present results for a 2 µm × 0.5 µm rectangular hole. Figure 3a shows η predicted by FDTD as a function of angle of incidence for this hole. Figure 3b shows the measured near-field profile for θ = 15°. For ease of visualization in Fig. 3c we have plotted the intensity on both sides of the holes along the dashed lines in Fig. 3b. The measured *η* for θ = 15° is approximately 4, compared to 4.1 from FDTD simulation. Figure 3d,e are near field measurement results for θ = 42°. The signal in the left side of the hole in this case is very close to the noise level, and as a result it is not possible to measure the exact value of *η* from these measurements. However, these results confirm the predictions from FDTD that the directionality of SP excitation is much higher for θ = 42° than compared to θ = 15°.

## Applications of the proposed excitation scheme

The proposed SP excitation scheme is simple to implement and can provide very high degree of unidirectionality. In the following, we present two examples to illustrate its usefulness: local excitation of plasmonic waveguides and surface plasmon nanolithography.

### Local excitation of plasmonic waveguides

**Figure 4:**
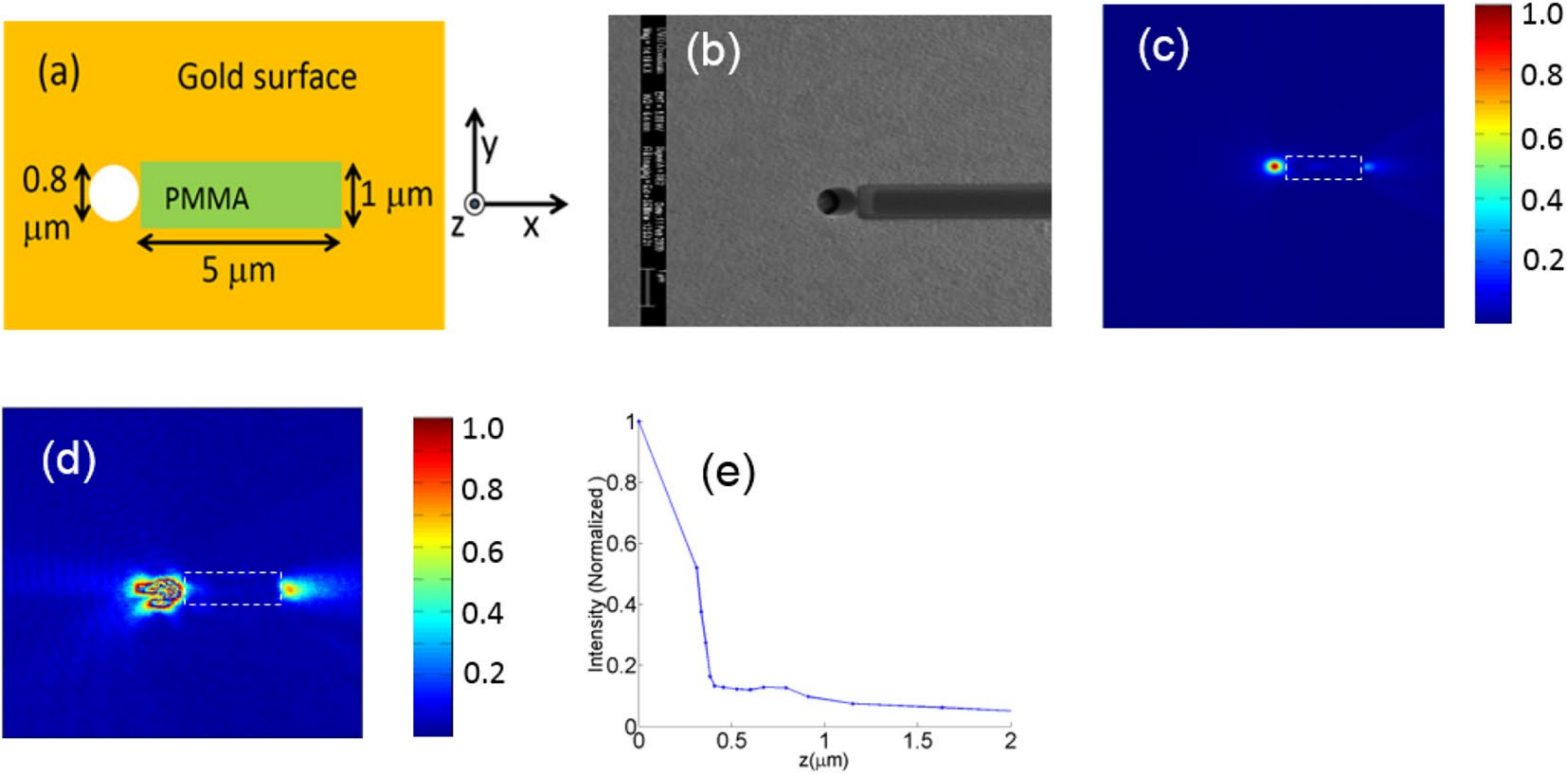
(**a**) Proposed structure. (**b**) Fabricated sample. (**c**) Intensity above the sample surface calculated using FDTD. (**d**) NSOM measurement results. (**e**) Intensity measured along the vertical direction (*z*-axis) after the end of the waveguide.

**Figure 5:**
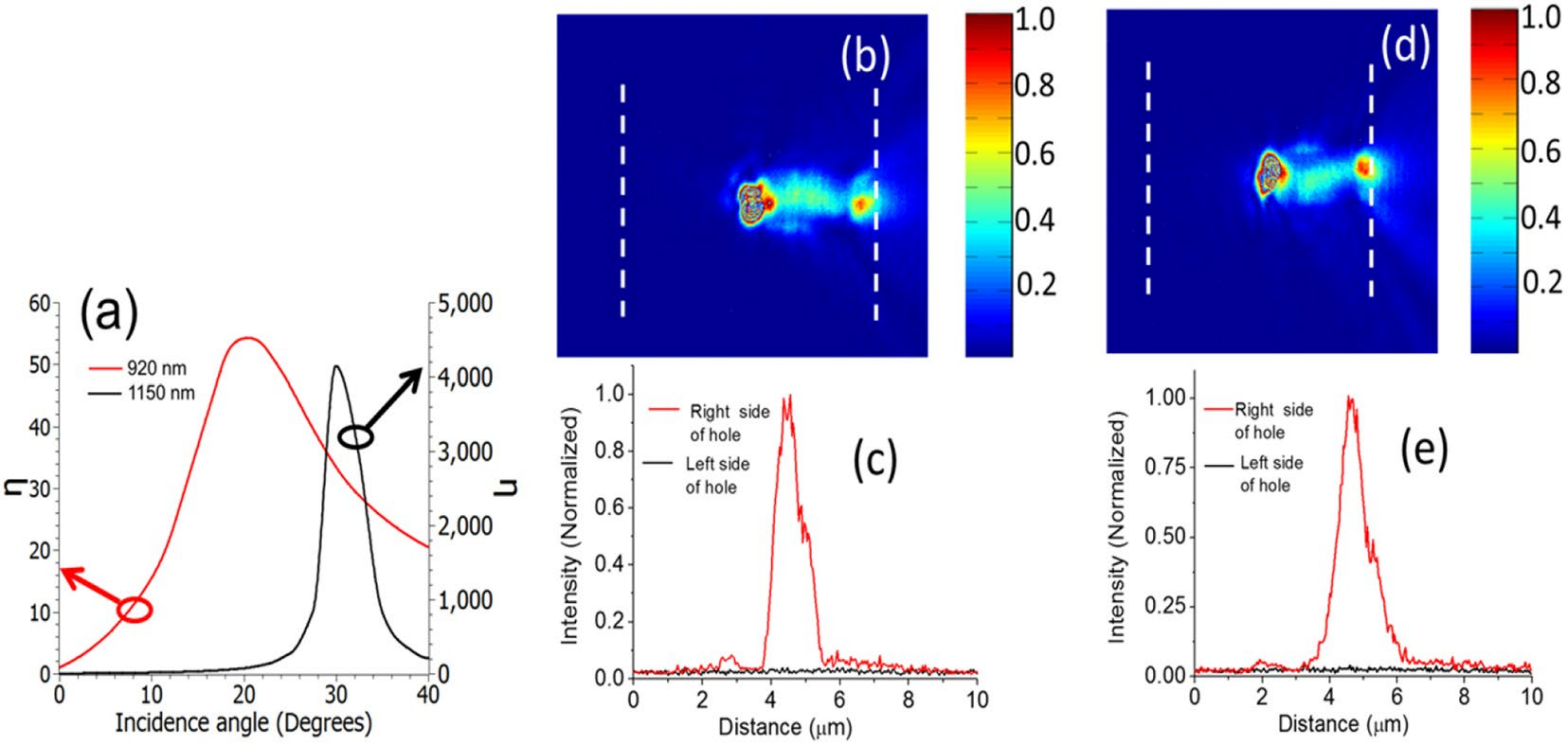
(**a**) Unidirectionality for various tilt angles for the structure shown in Fig. 4a. (**b**) and (**c**) Near field measurement results for θ = 22°. (**d**) and (**e**) Near field measurement results for θ = 37°.

To illustrate the usefulness of the proposed excitation scheme for plasmonic waveguides, we fabricated and tested the structure shown in Fig. 4a. It consists of a PMMA waveguide only on one side of the hole. This allows us to compare the light intensity at two sides of the hole and estimate unidirectionality. Figure 4b shows the fabricated structure. It consists of a circular hole with 800 nm diameter milled in 200 nm thick gold film. The hole is adjacent to a 5 µm long and 300 nm thick PMMA line. Figure 4c shows the near field profile calculated by Lumerical FDTD for light of 920 nm wavelength propagating along the z axis and illuminating the hole from beneath the metal surface. Electric field for the illumination is aligned along the y direction. Figure 5d shows the measured near field profile on the metal surface for the same illumination condition. Because of the finite size of the NSOM tip (200 nm diameter), the optical resolution is moderate as expected^35^. The good agreement between the FDTD results and the NSOM measurement is evident from Fig. 4c,d. To verify that light exiting the hole is indeed converted to SP, we measured the light intensity along the vertical (*z*) direction. The field confinement 0.5 μm after the end of the waveguide is shown in Fig. 5e. The field has higher confinement at the metal surface, and decays as we move away from metal surface indicating that the PMMA waveguide is successful in converting power exiting the slot into the SP mode. In addition to the simulation and experimental results presented in this section, we also present a simplified theoretical model in the Supplementary Information section, which provides insight into the effect of dielectric film thickness on the surface plasmon excitation efficiency.

The unidirectionality measured for normal incidence case is approximately 8.7, which is comparable with previous reports^31^. Higher level of unidirectionality would be required in many practical applications. This can be achieved by properly choosing the angle of incidence and having the PMMA guide, which will combine two effects to enhance unidirectionality: (a) sending most power to one side at the exist of the slot (by controlling the mode excitation by suitable choice of angle of incidence) and (b) by coupling light at the exit of the slot to SP (by proper choice of PMMA height). Figure 5a shows *η* as a function of tilt angle calculated using FDTD for the structure shown in Fig. 4a for two different wavelengths (920 nm and 1150 nm). These results illustrate that high unidirectionality can be achieved using this structure over a broad wavelength range. Figure 5b,c show the measured near field profiles at 920 nm wavelength for 22° angle of incidence. Figure 5d,e show the near-field profiles for 37° for the same sample. As expected from simulations, the SP excitation is highly unidirectional for both angles. The low power level on the left side of the hole makes it challenging to measure *η*, but the high levels of directionality for both angles are evident from these measurements.

### Surface plasmon interference lithography

**Figure 6:**
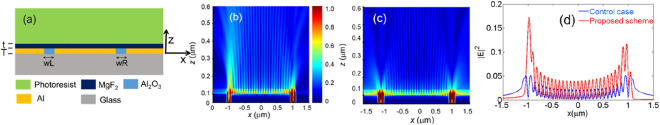
(**a**) Schematic of the proposed SPIL scheme. Dimensions are t = 30 nm, T = 100 nm, wL = 105 nm, wR = 140 nm. z = 0 is located at the metal-MgF_2_ interface, and x = 0 is the midpoint between the two slots. (**b**) and (**c**) FDTD results for square of electric field amplitude (|E|^2^) for proposed scheme and control case (**d**) (|E|)^2^ across a line 50 nm above the metal for tilted excitation (37° from z axis) shown by the red line. For comparison, |E|^2^ for the control case is shown by blue line.

Surface plasmon interference lithography (SPIL) is an emerging lithographic method capable of overcoming the diffraction limit of conventional photolithography^36–38^. The most important figure of merit of SPIL is the image contrast. Many previously reported SPIL schemes fail to achieve high image contrast, or achieve this by utilizing structures, which are challenging to fabricate. Previously we explained that the direction of SP can be controlled by adjusting geometrical parameter and angle of illumination. Here we use this principle to design a SPIL scheme for deep ultraviolet lithography to overcome some of the limitations of previous proposals. As shown in Fig. 6a, the proposed structure consists of two slots in a 100 nm thick aluminum film. The slots are filled with alumina, and the entire structure is coated with a 30 nm thick magnesium fluoride (MgF_2_) layer. The presence of the MgF_2_ film leads to large light intensity near the metal surface similar to conductor gap mode^39^ and provides high light intensity near the metal surface. We assume that a semi-infinite photoresist having refractive index 1.7 fills the upper half space above the mask. The width of the left and right slots are 105 nm and 140 nm respectively. These dimensions are chosen to ensure that, under oblique illumination, the SPs exiting the holes will propagate towards each other, and create an interference pattern between the slots. Figure 6b shows the square of the electric field amplitude (|E|^2^) across the structure when it is illuminated from the bottom at 37° with respect to normal to the metal surface at 260 nm wavelength. The SP generated by the left and right hole propagates toward each otherand form a standing wave pattern between the slots. Figure 6c shows |E|^2^ for a control case. The structure for the control case is identical to that shown in Fig. 6a with the exception that both slots are 140 nm wide. The illumination is normal to the surface in that case. Figure 6d shows |E|^2^ at a distance of 50 nm above the metal film for the proposed structure (red line). For comparison |E|^2^ for the control case for the same location is also shown in the same figure (blue line). The half pitch of the interference pattern is 36 nm—much smaller than achievable by conventional photolithography. As shown in Fig. 6d, the proposed scheme provides larger contrast in the region between the two slots (which we will call “slot region”) than the control case, which will make it useful for a wider range of photolithography processes. For the control case, the field intensity is significant outside the slot region. This field may result in formation of unwanted interference pattern outside the slot region, if any surface feature is present nearby. In contrast for the proposed scheme, the electric field strongly decays outside the slot region, and the chance of interference patterns forming outside the slot region is greatly reduced. The simplicity of the proposed scheme, its ability to provide large contrast, and highly localized field profile will extend the applicability of SPIL for nanofabrication.

## Conclusions

We have proposed and experimentally demonstrated that oblique backside illumination can be a simple method for achieving unidirectional SP excitation. We show that light can be coupled to a dielectric loaded plasmon waveguide with high directionality by using this simple geometry. The directionality we achieved is better than many previous proposals that uses a more complicated geometry. Because of the limitations of previously reported local SP excitation schemes, far field excitation is still commonly used in nanoplasmonics^24–29^. We believe the simplicity of this scheme will make it a very suitable choice of excitation for many widely used plasmonic waveguides^40^. We also present the design of a simple scheme of surface plasmon interference lithography capable of providing very large image contrast. We believe the simplicity, and the effectiveness of the proposed scheme will make it a widely used method for many applications including plasmonic sensing^41^, read/write operation in optical storage medium^42^, and controlling the near field interactions in plasmonic random metasurfaces^43^.

## Methods

### Sample preparation

The gold films were supplied by Ssens Bv. The sample consists of a 200 nm thick gold film with a thin titanium adhesion layer deposited on a glass substrate. A number of isolated holes were milled on the gold surface using a focused ion beam. The structure shown in Fig. 4b was fabricated as follows. Holes were milled with a focused ion beam in a 200 nm thick gold film on a glass substrate. For the fabrication of the PMMA waveguide, 950 PMMA A3 was spun on the sample and the pattern was defined using a Vistec 5000 + electron beam lithography system and developed using a MIBK:IPA developer solution.

### Measurement set up

300 mW input power from a Ti-Sapphire laser at 920 nm wavelength was coupled through the hole by a series of optical components. In the beam path two polarizers were used. By keeping the second polarizer at a fixed angle, and rotating the first one, we controlled the power incident on the sample, while linear polarization is maintained. To avoid over heating of sample, and possible damage to the PMMA waveguides we use a relatively large spot size (diameter 7 mm). The angle of incidence of light to the sample is controlled by using a tilted mirror. The spot size of the illumination is approximately 100 μm. The near field images were recorded using Multiview‐100 near field scanning optical microscope working in the collection mode to measure the near field on top of the metal surface. Each point in the NSOM images is collected with 10 ms average time. Each frame is 256 by 256 pixels.

## Supplementary information

Supplementary Information.
