# Supplementary material for: Directional excitation of surface plasmon using multi-mode interference in an aperture
Source: Sci Rep. 2021 Feb 4;11:3170. doi: 10.1038/s41598-020-78594-7 (PMC7862445; doi:10.1038/s41598-020-78594-7)
Supplement: Supplementary file 1 — Supplementary Information. [file 41598_2020_78594_MOESM1_ESM.docx]

**Supplementary Information**

Directional Excitation of Surface Plasmon using Multi-mode interference in an Aperture

M. Z. Alam*^1^, Z. Yang^2^, M. Sheik-Bahae^2^, J. S. Aitchison^3^ and M. Mojahedi^3^

1 Department of Electrical and Computer Engineering, Queen’s University, Canada, K7L 3N9

2 Department of Physics and Astronomy, University of New Mexico, Albuquerque NM, 87131, USA

3 Department of Electrical and Computer Engineering, University of Toronto, Canada, M5S 3G4

KEYWORDS: surface plasmon, plasmonics, subwavelength optics.

Corresponding author: [m.alam@queensu.ca](mailto:m.alam@queensu.ca)

We present a simple theoretical model which provides insight about the effect of dielectric film thickness on the surface plasmon (SP) coupling efficiency in case of illumination of a slot in a metal film (Fig. S1(a)). SP is the dominant field component of the near field in the vicinity of a metal nanostructure in the visible wavelengths, but radiation modes become increasingly more dominant for longer wavelengths [1]. Therefore, we carried out a theoretical analysis to confirm that SP can be excited efficiently with our structure working at near infrared wavelengths (920 nm). As discussed by Ung et al. [2], a slot in a metal film can be modeled as a horizontal Hertzian dipole immediately above the metal surface. This model, while simple, provides important physical insight into the excitation of SP from a slot and has shown excellent agreement with experimental results. According to this model we represent the structure shown in Fig. S1(a) by the one shown in Fig. S1(b).


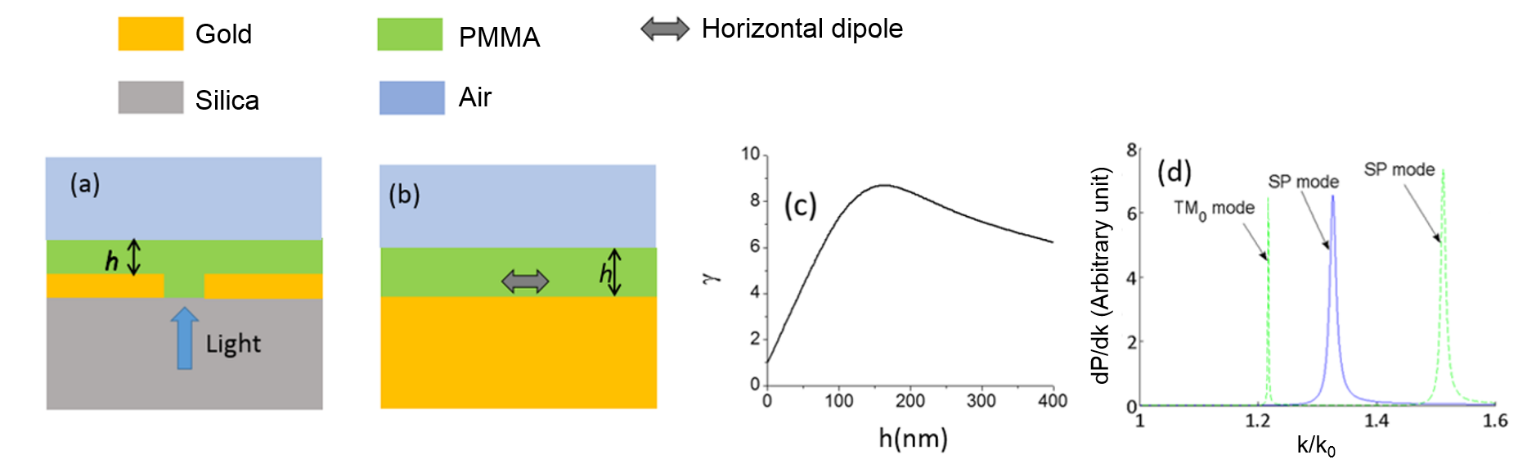


Fig. S1 (a) Excitation of SP through metal slot. (b) Equivalent model for the structure shown in (a) where the slot is replaced by a small horizontal dipole. (c) Enhancement (γ) as a function of PMMA thickness for the structure shown in (b). (d) Power spectrum of dipole emission. The solid line and dashed lines are for h = 150 nm and h = 350 nm respectively. Wavelength of operation is 920 nm.

To examine the effect of PMMA thickness on the SP conversion efficiency, we define the enhancement in SP excitation (γ) as

| $\gamma=\frac{P_{SP}^{PMMA}}{P_{SP}^{Air}}$ | (2) |
| --- | --- |

Here, $P_{SP}^{PMMA}$is the fraction of power radiated by the dipole embedded in the PMMA film (Fig. S1(b)) that couples to the SP mode. $P_{SP}^{Air}$ is the same quantity when the PMMA film is not present. For a bare metal, a significant amount of power is lost as radiation, and only a fraction of the light is converted to SP mode. In the presence of the PMMA film, there is better phase matching between the near field of the dipole radiation and SP. This results in an increased value of γ (Fig. S1(c)). For large film thicknesses, the phase matching degrades, and the value of γ starts to decrease again. Figure S1(d) shows the power spectrum calculated for two different PMMA thicknesses (h) using the procedure outlined in [3]. Here P, k and k_0_ stands for power, propagation constant and free space wave number respectively. The areas under the curves are proportional to the power coupled to the various modes. For h =150 nm, the spectrum has only one peak which is centered at k/k_0_ and corresponds to the effective mode index of SP supported by gold-PMMA-air for this PMMA thickness. For larger film thickness (e.g. h = 350 nm) the power spectrum, shown as the green dashed lines, exhibits two peaks. The values of k/k_0_ for these peaks correspond to the effective mode indices of the TM_0_ mode and the SP mode for this PMMA thickness. Since the power emitted by the dipole is split between the SP and dielectric waveguide mode for the thicker PMMA film, efficiency of SP excitation (γ) is reduced, as shown in Fig. S1(c).

1. Chen, L., Robinson, J. T. & Lipson, M. Role of radiation and surface plasmon polaritons in the optical interactions between nano-slit and a nano-groove on a metal surface. Opt. Exp. 14, 12629–12636 (2006).

2. Ung, B. & Sheng, Y. Optical surface waves over metallo-dielectric nanostructures: Sommerfeld integrals revisited. Opt. Exp. 16, 9073–9086 (2008).

3. Ford, G. W. & Weber, W. H. Electromagnetic interactions of molecules with metal surfaces. Phy. Rep. 113, 195–287 (1984).
